# How Green Are the Streets Within the Sixth Ring Road of Beijing? An Analysis Based on Tencent Street View Pictures and the Green View Index

**DOI:** 10.3390/ijerph15071367

**Published:** 2018-06-29

**Authors:** Rencai Dong, Yonglin Zhang, Jingzhu Zhao

**Affiliations:** 1 State Key Laboratory of Urban and Regional Ecology, Research Center for Eco-Environmental Sciences, Chinese Academy of Sciences, Beijing 100085, China; dongrencai@rcees.ac.cn (R.D.); ylzhang_st@rcees.ac.cn (Y.Z.); 2 College of Resources and Environment, University of Chinese Academy of Sciences, Beijing 100049, China

**Keywords:** green view index, Tencent street view, visual greenery, street greening, landscape assessment

## Abstract

Street greenery, an important urban landscape component, is closely related to people’s physical and mental health. This study employs the green view index (GVI) as a quantitative indicator to evaluate visual greenery from a pedestrian’s perspective and uses an image segmentation method to calculate the quantity of visual greenery from Tencent street view pictures. This article aims to quantify street greenery in the area within the sixth ring road in Beijing, analyse the relations between road parameters and the GVI, and compare the visual greenery of different road types. The authors find that (1) the average GVI value in the study area is low, with low-value clusters inside the third ring road and high-value clusters outside; (2) wider minor roads tend to have higher GVI values than motorways, major roads and provincial roads; and (3) longer roads, except expressways, tend to have higher GVI values. This case study demonstrates that the GVI can effectively represent the quantity of visual greenery along roads. The authors’ methods can be employed to compare street-level visual greenery among different areas or road types and to support urban green space planning and management.

## 1. Introduction

Urban greenery can improve neighbourhood and landscape aesthetics and enhance human psychological well-being [1,2]. Green vegetation not only regulates urban microclimates, sequesters carbon, releases oxygen, reduces noise and absorbs harmful gas but can also relieve psychological stress and improve the mood of urban residents [3,4,5,6,7,8,9]. To measure the quantity of urban greenery perceived by people, Aoki first advanced the concept of the green view [10], which was defined as the proportion of green vegetation in a pedestrian’s field of view. Ohno [11] demonstrated that visual greenery can alleviate the negative visual impact of artificial buildings. Urban visual greenery plays an important role in promoting the attractiveness and walkability of a neighborhood [12,13,14]. The existence of visible greenery can increase people’s rating of urban scenes [15,16]. High-resolution remote sensing images and oblique photographs were widely used in past studies of urban greenery to estimate the spatial distribution of green vegetation on the ground [17,18]. However, people at ground level actually see the profile of the landscape, which is not necessarily well-represented by the area of land covered by green vegetation [19,20]. Recent studies have demonstrated that pictures of real scenes can act as alternative data sources [19] and that image processing algorithms can be adopted to objectively identify and extract green vegetation polygons from the pictures to estimate the quantity of perceived greenery [20].

The indicator, “street greenery acreage” has been used to represent the general situation of horizontally distributed street greenery in traditional urban greenery assessments in China, but this indicator cannot fully represent what people at ground level truly see and is, therefore, unsuitable for use in the estimation of the abundance of resources and the layout of urban street greenery. Street view pictures (SVPs) show profiles of the landscape from a pedestrian’s perspective, and the Green View Index (GVI) can quantify the amount of greenery within a pedestrian’s field of view, which can partly make up for the deficiencies of the traditional assessment indicator. Based on previous studies, the GVI values were calculated using a large number of Tencent street view (TSV) pictures and the GVI formula. Taking the area within the sixth ring road of Beijing as a case study, the spatial distribution characteristics of urban visual greenery were analysed at both the point and street level to assess the urban greenery. The urban greenery values of different road types were compared to identify differences at the street level. The correlations between road parameters and GVI values were explored, and areas and locations in urgent need of improvement are identified. Moreover, different GVI calculation methods are compared and discussed. The main objective of this study is to provide a data processing tool to quantify street visual greenery on a large scale and verify its usefulness.

## 2. Literature Review

### 2.1. Using Street View Pictures in Urban Studies

Street View is a kind of interactive electronic map that provides 360° panoramas along urban streets or blocks for all users. Google, Tencent and Baidu are the three most representative street view mapping service providers in the world, and in addition to releasing street view map services for user browsing, they have released application program interfaces (APIs) for developers to customize web applications. As representations of urban landscapes, street view maps provide considerable image resources for urban visual greenery research and have the advantages of high accessibility, high resolution and broad coverage. SVPs have been used to study the relationship between psychological perception and the quantity of visual greenery. The crowdsourcing dataset, “Place Pulse” was used to explore whether green vegetation and its distribution had an impact on pedestrians’ feelings of safety, and the experimental results showed that green vegetation higher than 2.5 m tends to have a positive impact on people’s perceived safety [21,22]. Yin et al. [23] provided an automatic pedestrian detecting-and-counting tool based on Google street view (GSV) picture datasets and machine learning algorithms to assist local road planners and designers to increase road walkability in three American cities (Buffalo, NY, Washington, DC, and Boston, MA). Liu et al. [24] provided a practical tool to measure physical qualities at street level in Beijing, China, based on GSV pictures and machine learning models. Seiferling et al. [25] used enormous SVP datasets to measure perceived tree crowns along roads in Boston and New York City, U.S.A., to better understand the presence and spatial distribution of vegetation from a pedestrian’s perspective. Those studies showed that vast SVP datasets area useful quantification tool for helping decision-makers, planners and researchers to understand street-level landscapes from a pedestrian’s perspective.

### 2.2. Using the Green View Index to Quantify Visual Greenery

In early green view studies. researchers took pictures at specific locations after sampling sites were chosen and then used Adobe Photoshop software to extract green vegetation from those pictures to calculate the green view index (GVI) values [19]. Yang and colleagues demonstrated that the GVI value was affected by the size of the tree crown and the distance between the photographer and the subject [19]. However, the process of taking pictures on site is time consuming, easily affected by weather conditions and difficult to use to conduct numerous urban greenery evaluations. Moreover, the GVI values between different districts were difficult to compare. Li improved upon the previous method, using GSV pictures as the data source and using an image segmentation algorithm based on colour classification to extract green polygons from the pictures. The experimental results in this study showed that the calculation method was accurate and effective [20].

The image processing technique improves the quantification of landscape components in SVPs and the calculations are simpler and more precise. Liang et al. [26], Shen et al. [27] and Zhang and Dong [28] adopted computer vision methods based on SegNet to segment and quantify the proportion of visible landscape elements in an area using SVP datasets. Gong et al. [29] adopted another pixel-level semantic segmentation method, namely, the Pyramid Scene Parsing Network (PSPNet), to extract sky, trees, and buildings from a panoramic street view picture dataset in Hong Kong, China. These studies indicated that image processing algorithms can be excellent tools for helping urban planners, decision-makers and the public to better understand streetscapes from a pedestrian’s perspective.

The relevant literature shows that a strong correlation exists between urban visual greenery and social-economic factors. Based on their previous works, Li et al. further explored the correlation between urban greenery and residential (economic and racial) status in Hartford, Connecticut and found that residents with higher incomes tend to live in urban areas with more visual greenery [20]. Moreover, the GVI has been used to evaluate the inequity of residential green space. Li et al. found that at the block level, residents had unequal accessibility to different types of urban greenery (street greenery, private yard total vegetation, private yard trees/shrubs and urban parks), providing a foundation for studying urban greenery inequity [30]. The indicator GVI has been generalized for application to a wide variety of urban green space. Yu proposed the floor green view index (FGVI) based on the GVI and demonstrated through experiments that this indicator can be used in the evaluation and planning of high-rise building construction projects [31]. The FGVI can be used to evaluate urban greenery visibility inside high-rise buildings from the perspective of the occupants. However, due to strategic and business policies, Google has not provided the Google street view (GSV) service in China. Therefore, Tencent street view (TSV) pictures have been used in GVI studies in Chinese cities as a substitute for GSV images. Long and Liu calculated the GVI values of the central areas (a circular area with a 3-km radius) in 245 Chinese cities and compared the overall GVI conditions of these cities. The results showed that the GVI rankings of these cities were consistent with the official evaluation criteria of the “Garden City of China” [32]. Cheng et al. [33] measured multiple visual attributes of streets in the Jianye district of Nanjing city, China, including the salient region saturation, visual entropy, GVI, and sky-openness index. A recent study proved that street-visible greenery has a positive effect on housing prices in Beijing. Zhang and Dong (2018) adopted an extensive street view picture dataset and a horizontal green view index (HGVI) to quantify the street-visible greenery neighbouring the housing estates within the fifth ring road region in Beijing, China. 

## 3. Materials and Methods

In this section, the study area is introduced and then the authors demonstrate, in detail, the GVI calculation procedure. The urban street GVI calculation procedure includes several steps: first, simplifying the Beijing road network to centre road lines, and establishing equidistant road site sampling locations (at intervals of 100 m) along the centre road lines; second, using a crawler written in Python to download the nearest TSV picture at all sampling sites (with a maximum search radius of 50 m); third, taking an image segmentation algorithm based on the hue, saturation and intensity (HSI) colour model to extract the green vegetation polygons in TSV pictures; and finally, calculating the GVI values of all sites via the GVI calculation formula.

The analysis processes and methods are as follows. First, the overall condition of urban street greenery in the study area is analysed. Second, the GVI values of different road types are compared. Third, the relationships between GVI values and road parameters (the length and width of streets) are analysed. Fourth, the various GVI calculation methods and their results are analysed and discussed.

### 3.1. Study Area

**Figure 1:**
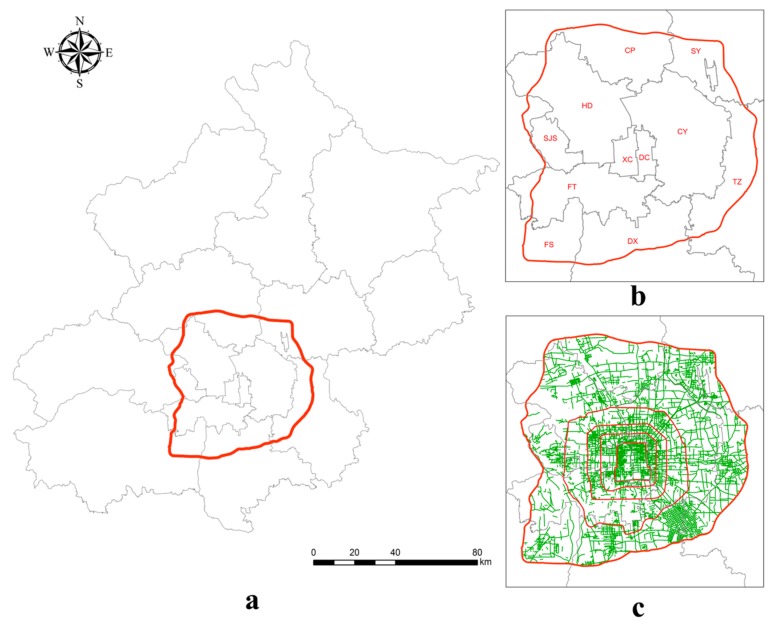
Study area in Beijing city. XC: Xicheng district, DC: Dongcheng district, HD: Haidian district, CY: Chaoyang district, CP: Changping district, SY: Shunyi district, TZ: Tongzhou district, DX: Daxing district, FS: Fangshan district, FT: Fengtai district, SJS: Shijingshan district. Subplot (**a**) shows the location of the sixth ring road area. Subplot (**b**) is a part of the administrative division map in Beijing. The red lines in (**c**) from inside to outside separately represent the second, third, fourth, fifth and sixth ring roads in Beijing.

Beijing is the political, financial and cultural centre of China and has approximately 21.705 million permanent residents and an area of 16,410 km^2^. Twelve of the sixteen administrative districts lie partially or entirely within the sixth ring road area. The sixth ring road area (including the sixth ring road) has a total area of 2267 km^2^ and includes most of Beijing city proper and a small portion of the urban-rural junction. The research area in this study is shown in Figure 1a. The abbreviation for each district is shown in Figure 1b. The spatial distribution of street sampling sites is shown in Figure 1c. The sub-areas are surrounded by the sixth ring road and divided by the boundaries of the administrative districts, thus some of the sub-areas represent only part of the entire district. “HD”, for example, represents the portion of Haidian district lying within the sixth ring road.

### 3.2. Simplifying the Urban Road Network and Extracting Sampling Sites

**Figure 2:**
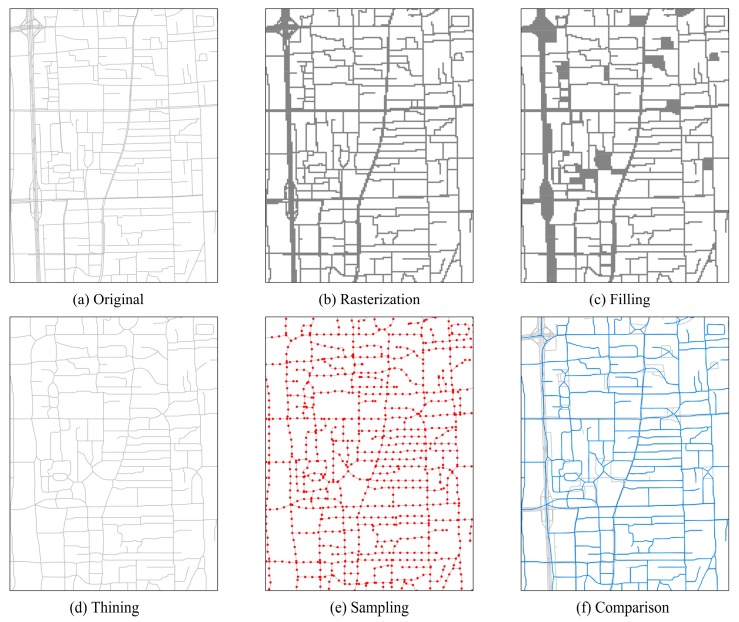
Road network simplification procedure.

The road system of Beijing was obtained from Navigator Company, the main navigation service provider in China. These road data, in vector format, were from the first quarter of 2013. The simplification processes of the road data comprised the six steps shown in Figure 2, and each step is explained below:

(a) Selecting the original roads. This paper selected the main roads of Beijing as the original roads. These roads included all motorways, urban expressways, major roads, national roads, provincial roads, and contained several minor roads and county roads with names.
(b) Rasterization. The original roads data were converted to raster format with a 20-m resolution.
(c) Filling. During the simplification of the original roads, the gaps around crossroads needed to be filled. This study used the ArcScan model in ArcGIS to fill the gaps. This process was used to produce a skeletal representation of the roads.
(d) Thinning. The thinning tool in ArcScan was used to simplify the roads after the filling process.
(e) Sampling. Following the thinning process, sampling spots were established every 100 m along the simplified roads.
(f) Comparison. The blue polylines shown in Figure 2 represent the simplified roads after performing steps (a) to (d), whereas the grey polylines represent the raw original road data.

Additionally, central polylines (simplified roads) were aggregated from multi-lane roads, and the road attributes, such as length and width, of multi-lane roads were averaged. Finally, these simplified road data with sampling spots produced by these processing steps were used in the TSV image acquisition process.

### 3.3. Obtaining TSV Pictures Based on Road Sampling Sites

**Figure 3:**
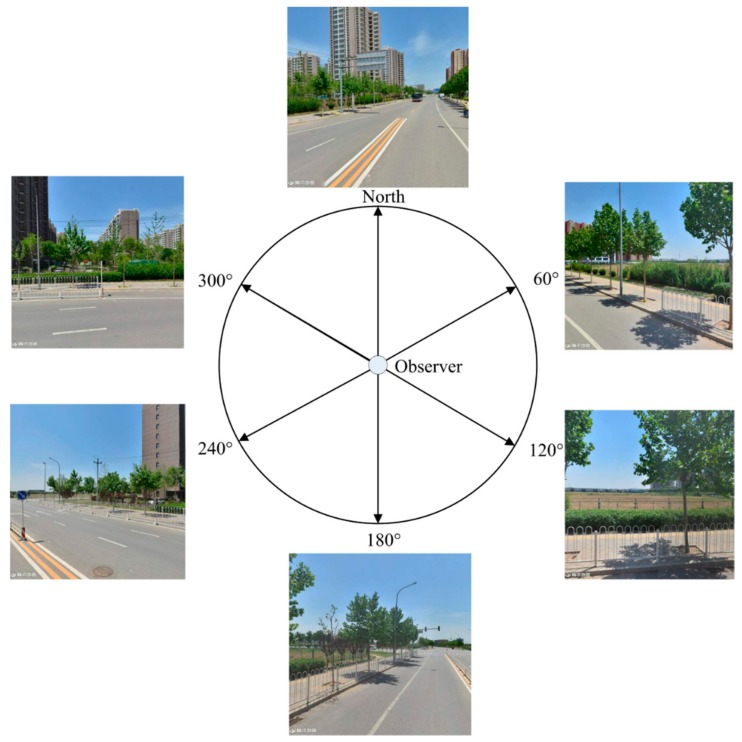
The configuration of Tencent street view (TSV) pictures.

The current study focused only on TSV pictures at the horizontal level and did not consider the vertical direction. The authors used six horizontal TSV pictures at the same interval angles to represent the visual landscape at each street sampling site (see Figure 3). The panoramic camera was about 2.5 m above the ground. The panoramic camera comprised 6 sub-cameras, including 5 facing in horizontal directions with a 72° intersection angle and 1 sub-camera facing in a vertical direction. The resolution of a TSV picture in this study was 600 × 600 and the format was PNG. Since the street view APIs of Google, Tencent or Baidu are similar, the method used in this article can be translated to other street view platforms. To avoid violations of privacy and personal information security, all faces and vehicle license numbers in TSV pictures were obscured via mosaic processing. Additionally, the authors checked the user license of the Tencent location service carefully to ensure that the software development obeyed the official protocol.

**Figure 4:**
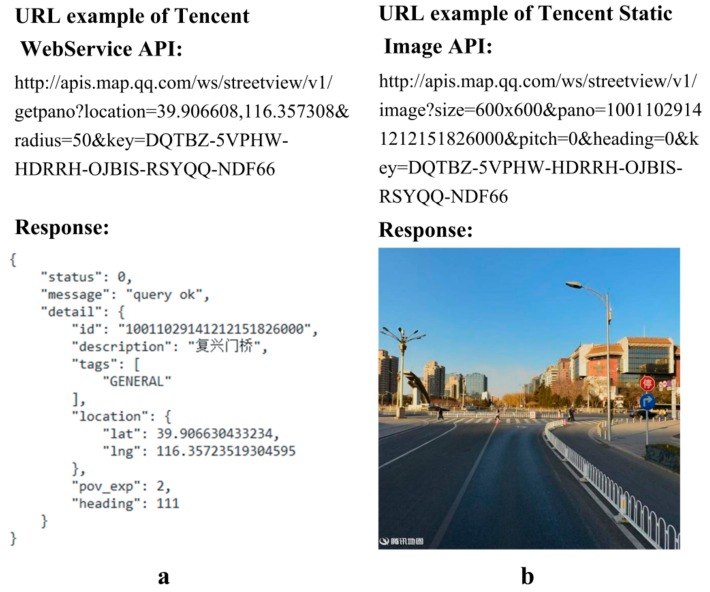
(**a**) uniform resource locator (URL) response of Tencent WebService and (**b**) static image application program interface (API).

The procedure for obtaining the TSV pictures at road sampling sites involved two steps: (1) finding the nearest TSV ID within 50 m based on the Tencent WebService API, and (2) using the Tencent static image API to obtain the six TSV pictures in the horizontal direction at the sampling site. The request format of those two Tencent API is URL (Uniform Resource Locator). The TSV picture acquisition at all sampling sites required the looped execution of the two steps and produced a total of 333,234 TSV pictures for 55,539 sampling sites. The URL request example and result at the coordinates 39.906608° N, 116.357308° E are shown in Figure 4a which shows the metadata received after sending a Tencent WebService URL request, and Figure 4b shows an example of a TSV picture obtained by sending a URL request to the Tencent server. The parameter lists and instructions of those two APIs, as well as the time–cost analysis of the parallel crawler, are described in the Appendix.

### 3.4. Extracting Green Vegetation from TSV Pictures

The authors developed an image segmentation script for automatically extracting green vegetation polygons from TSV pictures under the environment of MATLAB. The basic working principle of the segmentation script involved using the HSI model to limit the hue component (75 < Hue < 170) in a picture to obtain a mask, then used the mask to screen green pixels from the TSV pictures. The confirmation of the hue threshold extent was accomplished by optimizing the hue value to maximize the linear regression coefficient between the segmentation value and the manual segmentation value. Based on the HSI colour model, the green pixels were initially located in the thresholds 60–180, which were the initial thresholds in the parameter estimation experiments. The authors narrowed the thresholds on both sides to find a peak R-squared value and the corresponding thresholds were 75–170. Using the HSI colour model, the hue, saturation and intensity components were used to represent a true colour picture. The hue component identified the colour of each pixel; the saturation component identified the colour saturation of each pixel; and the intensity component identified the brightness of each pixel. The core calculation rule was shown in Table 1.

The hue mask in Step 4 was a binary matrix which recorded the pixel positions marked as green pixels. Then, a matrix manipulation filtered out the non-green pixels in the original street view picture.

### 3.5. Using TSV Pictures and the GVI Formula to Calculate GVI Values

The GVI formula for every sampling site is as follows:

(1) $$\mathrm{GVI}=\frac{\sum_{i=1}^{m}Area_{g\_i}}{\sum_{i=1}^{m}Area_{t\_i}}\times 100\%$$

Formula (1) uses *Area_g_i_* as the total number of pixels of green vegetation extracted by the image segmentation algorithm in direction *i*. *Area_t_i_* is the total pixel number in the whole picture in direction *i*. The parameter *m* is the number of pictures facing different directions in the horizontal plane; in this study, *m* = 6. The GVI calculation was adjusted based on the specific configuration of TSV pictures. Vertical-view GVI was calculated by adding SVPs in vertical or sub-vertical directions. A formula incorporating the upward direction would be more appropriate in a study of the relationship between tree crown density and GVI, for example. Therefore, based on the work of previous studies, the authors provide the general GVI Formula (2):

(2) $$\;{\mathrm{GVI}}_{g}\;=\;\frac{\sum_{j\;=\;1}^{n}\sum_{i\;=\;1}^{m}Area_{g\_ij}}{\sum_{j\;=\;1}^{n}\sum_{i\;=\;1}^{m}Area_{t\_ij}}\times 100\%$$

In Formula (2), *Area_g_ij_* represents the number of pixels of green vegetation extracted by the segmentation method in the horizontal direction *i* and the vertical direction *j*.

Similarly, *Area_t_ij_* represents the total area of the SVP in the horizontal direction *i* and the vertical direction *j*. Regarding Formula (2), the symbol “*g*” means “general”. Parameters *m* and *n* represent the numbers of horizontal and vertical directions, respectively. Considering Li’s GVI calculation formula, *m* = 6 and *n* = 3; hence, the number of SVPs at a single sampling site was 18 [20]. When *n* = 1 and the heading angle is 0 degrees, Formula (2) is equal to Formula (1). 

Additionally, when *m* = 1, *n* = 1 and a single image can represent the whole visual environment, the authors defined the GVI as the panoramic green view index (PGVI), as described by Formula (3):

(3) $$\;\mathrm{PGVI}\;=\;\frac{Area_{g}}{Area_{t}}\times 100\%$$

In Formula (3), *Area_g_* is the area of green vegetation in the panorama, and *Area_t_* is the total area of the panorama.

Selection of the most appropriate GVI calculation formulae depended on the configuration (horizontal and vertical directions and number of pictures) of the SVPs employed by the researchers. Section 5.3 will explore the differences among the calculation results of various GVI formulas.

## 4. Results

### 4.1. Green Vegetation Segmentation of TSV Pictures

**Figure 5:**
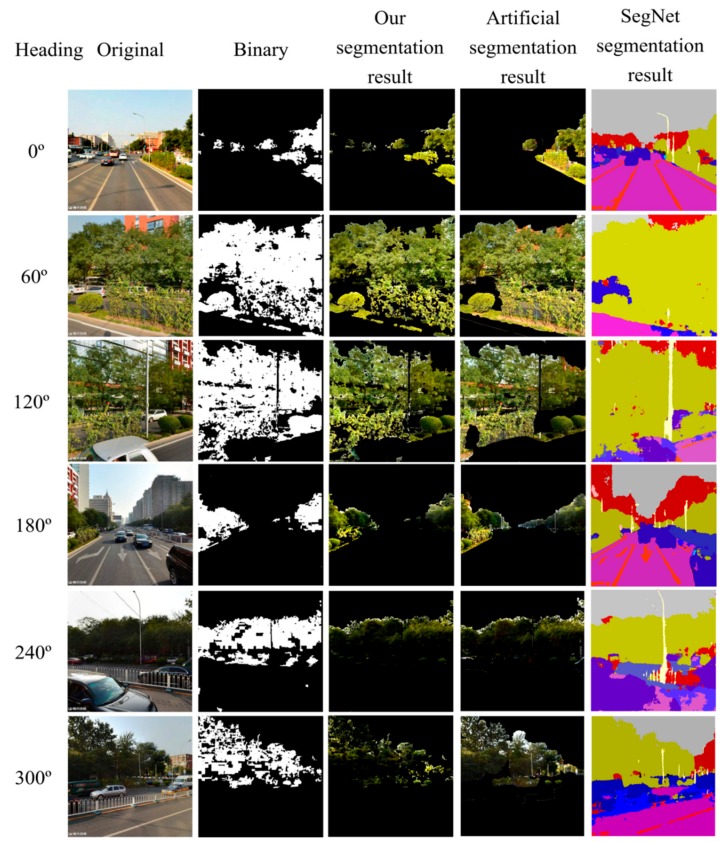
Comparison of segmentation results. Coordinates: 41.0574526° N, 116.635947° E.

Figure 5 shows the original TSV pictures and segmentation results at one random sampling site on Yuetan North Street in the XC district. The first column, in Figure 5, of the picture matrix shows the TSV pictures in the horizontal direction; the second column shows the binary mask obtained by the segmentation rule in Section 3.3; the third column shows the extracted green vegetation polygons using the binary mask from the original pictures; the fourth column shows the artificial segmentation results; and the last column is the result extracted by the pixel-level semantic segmentation method (SegNet). The GVI value of this sampling site is 0.288 based on Formula (1). 

### 4.2. GVI Accuracy Verification

**Figure 6:**
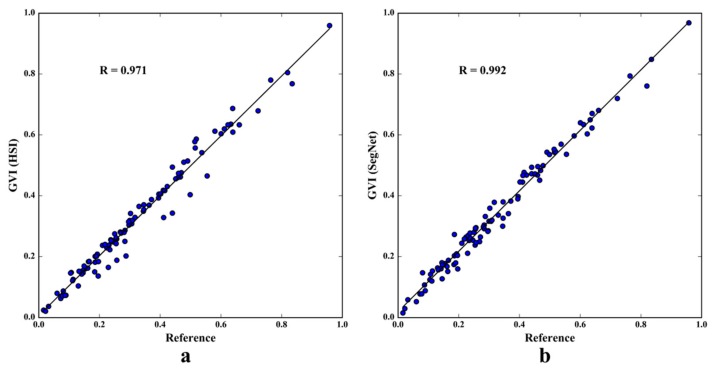
The accuracy inspection of green view index (GVI). Reference refers to the results of artificial segmentation; HSI (Hue, Saturation and Intensity) segmentation result refers to the GVI results of the authors’ algorithm; SegNet segmentation result refers to the GVI values calculated using the SegNet method.

To further verify the accuracy of the image segmentation, the authors extracted 100 random pictures in the TSV dataset and compared these segmentation results with the artificial Adobe Photoshop segmentation results and the SegNet segmentation results using linear regression. The black line in Figure 6 is the fitting line between the independent and dependent variables. Figure 6a shows that the regression line is very close to the diagonal line, and Pearson’s r = 0.971, with *p* = 0.000, shows that the independent and dependent variables have a strong correlation. Similarly, Figure 6b shows the fitting line between GVI values calculated by the current method and by SegNet, and Pearson’s r = 0.992, with *p* = 0.000, which means that the two variables have a significant positive correlation. The results show that the GVI values calculated by the current method are consistent with both the artificial extraction and the SegNet semantic segmentation. This verification of the GVI data indicates that the data can be used in the next analysis.

### 4.3. GVI Results at All Sampling Sites

**Figure 7:**
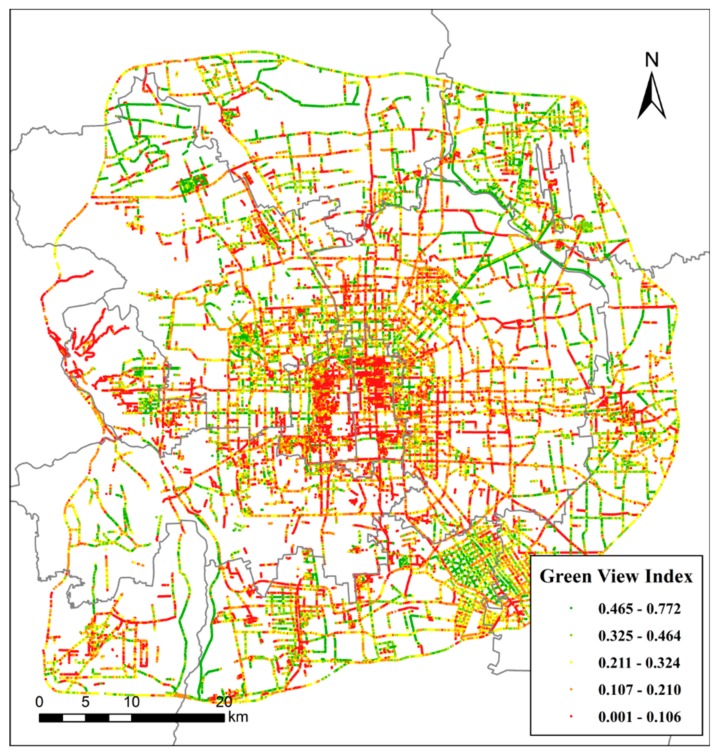
The green view index (GVI) values at sampling sites in the study area.

Figure 7 displays the entire GVI results at all sampling sites in the study area. The GVI values range from 0.01 to 0.772, with a mean of 0.171 and a squared deviation of 0.016. According to Aoki’s study [10], pedestrians felt more positive and comfortable at GVI values equal to or greater than 0.25. The quantity of urban street greenery in the current study area has room for improvement according to our GVI results and the 0.25 standard. The green elements along roads should be improved for pedestrians to achieve better, more relaxing streetscapes.

**Figure 8:**
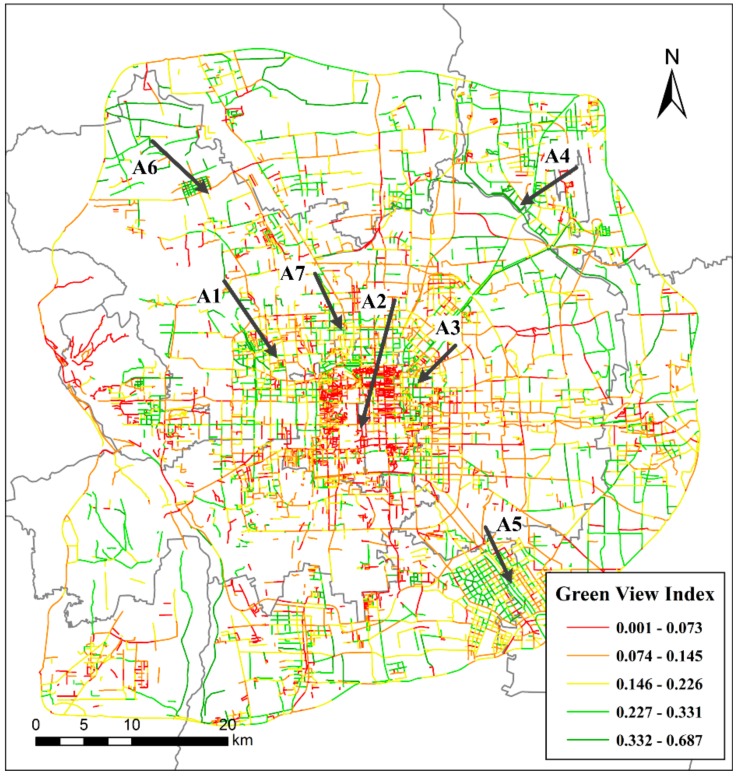
The green view index (GVI) values at street level. The GVI values have been aggregated from site-level GVI values in Figure 7. This map shows the spatial distribution of GVI at street level. Markers A1–A7 were used to identify the concentration areas of very high or low GVI in the study area.

The authors aggregated the GVI values of sampling sites for each road segment and the spatial distribution is shown in Figure 8. The GVI value at street level is the arithmetic mean of all GVI values along the road segment. The street-level data included road identification, road shape length, road shape width, road type and GVI after aggregating the point-level data. Based on the natural break method, the GVI values are separated into five intervals: very low (0.001, 0.073), low (0.074, 0.145), medium (0.146, 0.226), high (0.227, 0.331) and very high (0.332, 0.687). Taken from the centre of the study area, the GVI values on the west side adjoining the capital core area, which is composed of the DC and XC districts, are high (see marker A1). However, the GVI values to the east and south of the core area are very low (A2). Regarding the periphery of the core area of Beijing, the GVI values in the adjacent areas of CY and SY districts are high (A3 and A4). High GVI road segments are concentrated in the southern TZ district (A5). Additionally, high GVI road segments are also concentrated in the north-western and south-eastern parts of the HD district (A6 and A7).

The five ring roads are the most important urban expressways in Beijing and are beneficial for alleviating traffic congestion and driving peripheral economies In the study area, the five ring roads from the centre to the periphery—the second ring road to the sixth ring road—have GVI values of 0.17, 0.15, 0.12, 0.11 and 0.19, respectively. Therefore, from the second ring road to the fifth ring road, the GVI value at street level decreases from the centre toward the periphery. The GVI values of the second, third and sixth ring roads are classified as medium, and the values of the other ring roads are classified as low. Therefore, the fourth and fifth ring roads should be given more greening resources, not only to ensure that street greenery provides normal ecosystem functions but also to provide more street-visible greenery. Good street-visible greenery conditions could ease drivers’ anxiety and regulate their emotions [34], especially when they are in traffic jams, which are common in Beijing [35]. The GVI values of the areas between adjacent ring roads (excluding the GVI values of the ring roads themselves) are 0.09, 0.11, 0.16, 0.16, 0.15, and 0.20, which are classified as low, low, medium, medium and medium, respectively (the core of the study area is surrounded only by the second ring road). Therefore, for city managers and planners, the street-level visual greenery within the third ring road area are averagely low or medium and should be improved in the future, especially in the weak spots and segments marked on the GVI maps. 

### 4.4. Overall GVI Assessment in Study Area

**Figure 9:**
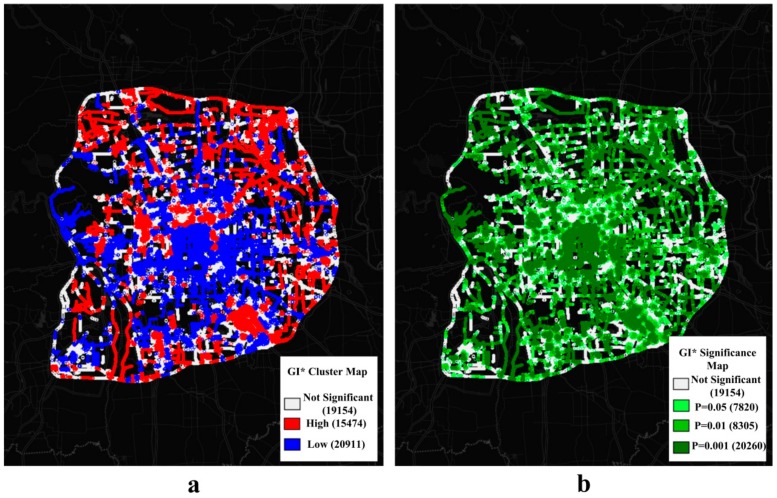
Getis-Ord GI* Cluster and Significance maps of green view index (GVI). Numbers of points are in parentheses. Subplot (**a**) shows the hot and cold spots of GVI in a GI* Cluster Map. Subplot (**b**) shows the GI* Significance Map of GVI values at 0.001, 0.01 and 0.05 level.

Figure 8 shows that the distribution of roads in the sixth ring area forms a radiating and lattice-like layout and that numerous intersections between the urban-rural expressways and the ring roads exist. The urban expressways and highways form connections to satellite towns. The authors calculated the Moran’s I value of all GVI values in the study area, with a result of 0.45 (*p* < 0.001), demonstrating that the GVI values have strong positive spatial autocorrelation. The Getis-Ord GI* tool in Geoda software was used to detect cold and hot spots in the study area. Additionally, the spatial weight matrix was calculated based on the Euclidean distance between sampling sites, based on a distance threshold set to 563 m. Figure 9a shows that the core region of the study area presents low-value clustering (cold spots), whereas the peripheral zone surrounding the core area mainly presents high-value clustering (hot spots). Figure 9b shows whether the spatial clustering of the sampling sites is significant and, if it is significant, at what level (0.001, 0.01 and 0.05 level). The core of the city has a high-density road network, and the points exhibit significant low-value clustering. The peripheral area surrounding the core area features significant aggregations of high GVI values, but the western SJS district and parts of the road segments in the west sixth ring road present significant low-value clustering. The analysis of spatial hot spots and cold spots can help urban planners understand the overall accumulation features of GVI and can, therefore, lead to more reasonable allocation and investment of afforestation resources. 

From the data, the authors found that the average GVI value had a significant positive correlation with the “street greenery acreage” in the districts in the study area (r^2^ = 0.77, *p* = 0.0012 (<0.01)). In 2014, the social statistical data of “street greenery acreage” was gathered from the official website of the Beijing gardening and greening bureau (http://www.bjyl.gov.cn/). The “street greenery acreage” data represents the area of green vegetation, including tree crowns, shrubs and grass along roads and is an indicator used to measure horizontal vegetation cover. Li et al. [20] demonstrated that tree crown cover and grass cover have a positive correlation with the modified GVI values for various radius buffers. The current results support those of Li’s study at the district level, demonstrating that at the district level, the roads with more horizontal street greenery tend to have higher GVI values.

## 5. Discussion

### 5.1. Comparing the GVI between Different Road Types

**Figure 10:**
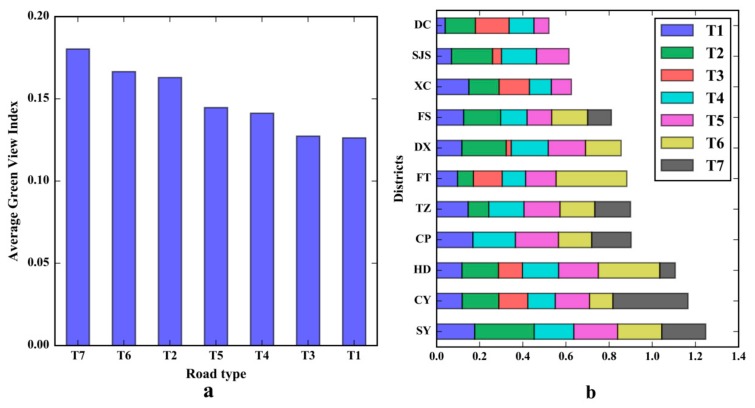
The statistical green view index (GVI) values of different road types. Subplot (**a**) shows a bar plot of the average GVI values in different road types. Subplot (**b**) shows the cumulative sum of the average GVI values in different road types.

There are seven major road types in the study area: motorways (T1), national ways (T2), urban expressways (T3), major roads (T4), minor roads (T5), provincial roads (T6) and county roads (T7). Based on the simplified road network data, the authors aggregated all sampling sites to 10,587 road segments in the study area (see Figure 8). The average road length is 347.9 m in the study area, and the average road width is 6.8 m. As shown in Figure 10a, T7 has the highest average GVI value of 0.180; whereas T1 and T3 have the lowest average GVI values of 0.126 and 0.127, respectively. Between these maximum and minimum average GVI values, the average GVI values of T6, T2, T5 and T4 are 0.167, 0.163, 0.145 and 0.141, respectively. Figure 10b is a stacked bar diagram, and the vertical axis shows the different districts (district names are in Figure 1b), and the horizontal axis shows the cumulative average GVI value of different road types. In Figure 10b, the different road types are represented by differently coloured bars, and the length of each bar represents the average GVI value (a longer bar represents a higher average GVI value). The DC district includes five major road types, T1, T2, T3, T4 and T5, with T3 having the highest GVI value among them, for example. The cumulative sum of the average GVI values can be used to evaluate the overall street greenery for all road types to determine the amount of greenery visible to pedestrians. These results have some reference value in terms of adjusting the road design of different road types and improving the amount of visual greenery available to people on the roads. More street-level visual greenery could improve the walkability of streets and enhance public mental health [8,36]. Based on the current data, the authors found that the road lengths of T1, T4 and T5 represented the majority (85.1%, 4891.5 km) of the total length of all road types. Moreover, these three road types exist in all of the districts in the study area. Therefore, T1, T4 and T5 should be considered for further greening design and planning tasks. Regarding T1, a central green belt with tall green vegetation and dense shrubbery could be adopted for further improvement of the street greenery design. Considering T4 and T5, growing taller trees with large crowns would be an effective way to increase visual greenery along the roads. The fundamental aspect of enhancing street visual greenery is increasing the profile area of green vegetation along roads. Previous studies have shown that Google Street View pictures can be low-cost and high-precision tools to measure street greenery at highly-motorized major roads in a western city [25,27]. The Tencent street view pictures dataset in this study offers quantification tools for a megacity with complex road types to quantify street visual greenery from pedestrians’ perspective.

### 5.2. The Relationship between the GVI and Road Parameters

The Pearson’s r^2^ values for the correlation between the GVI values and the road width and road length are 0.027 and 0.224, respectively, and are significant at the 0.01 and 0.000 levels in this study. Therefore, the street-level GVI has a significant positive correlation with road length at the global level but a low correlation coefficient with road width. Table 2 lists the Pearson’s r^2^ values and significance levels of different road types between the GVI and the road parameters (width and length). The GVI values of T1, T4 and T6 have negative correlations with road width and are significant at the 0.000 level, whereas the GVI values of T5 have a positive correlation with road width. Except for T3, the studied road types have significant positive correlations between GVI and road length to different degrees. 

To summarize, (1) the lengths and widths of roads are not always significantly correlated with GVI for all road types; (2) the GVI values of T1, T4 and T6 are significantly negatively correlated with road width, whereas those of T5 are positively correlated with road width; (3) road length is commonly a strong predictor of GVI values, but not for T3; and (4) the influence of road width on the GVI has a scale effect in the study area, with a weak influence at the global level and a stronger influence at the road type level. 

Therefore, based on the study of street-level GVI at the capital city level, the authors propose that road type should be an important parameter to consider in the analysis process due to the complexity of roads in a metropolis. Understanding the effects that road width and length have on GVI is helpful for designing and planning suitable green infrastructure along roads. Additionally, the current tools can accurately locate the weak spots according to GVI values and specific geographical locations where the low-GVI values are concentrated significantly. By further investing in the natural elements along those roads, the government could specify preliminary optimization solutions based on this study’s street view pictures, at a large scale, which contain the scene information.

### 5.3. Comparing Different GVI Calculation Methods

**Figure 11:**
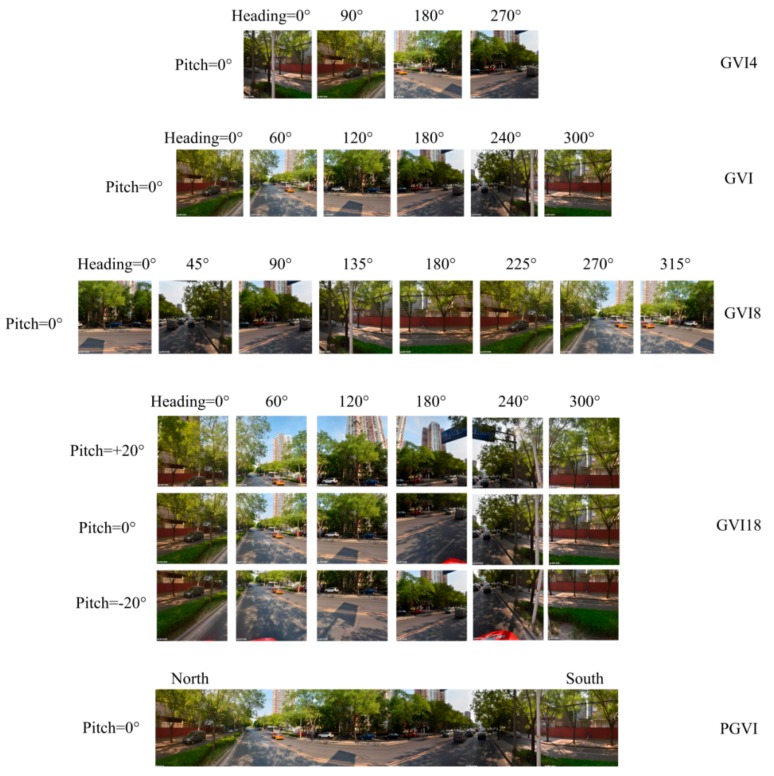
Five green view index (GVI) calculation methods and street view picture (SVP) configurations.

To date, there are no studies which compare and contrast GVI calculation methods, so the available results and calculation methods should be discussed and compared in detail. Figure 11 shows the configurations of different GVI calculation methods. To the right of each picture group, the GVI name of the specific configuration is shown. These specific configurations of SVPs are described below:

1. GVI4 represents the GVI value calculated from four horizontal TSV pictures, with a heading angle of 90° (see the picture group of GVI4 in Figure 11). Long and Liu [32] used this method to calculate GVI values.
2. GVI represents the result of the current method, in which six horizontal TSV pictures with a heading angle of 60° are used to obtain the GVI value (see the GVI picture group in Figure 11). Zhang and Dong [28] used this method to calculate street-visible greenery.
3. GVI8 represents the GVI value calculated from eight TSV pictures in the horizontal direction, and the heading angle is 45° (see the group of GVI8 in Figure 11).
4. GVI18 represents the GVI result calculated from six horizontal directions with a heading angle of 60° and three vertical directions with pitch angles of 30°, 0° and −30° (see the picture group of GVI18 in Figure 11). Li et al. used this configuration of SVPs to calculate the GVI value [20,30]. Due to the limitations of the pitch angle in the Tencent static image service (−20° to 90°), the authors actually used 0°, −20° and 20° as the pitch angles to simulate the calculation procedure of the method used by Li et al. [20]; hence, slight differences exist.
5. PGVI is calculated from a cylindrical panorama stitched together from six horizontal TSV pictures using Hugin software (see the PGVI group in Figure 11). Cheng et al. [33] adopted a similar method to pre-process the street view pictures.

The authors established four comparative groups, GVI and GVI4, GVI and GVI8, GVI and GVI18, GVI and PGVI. The authors’ method has been set as the reference. The average difference value and root mean squared error (RMSE) were the two indicators used to measure the degree of difference, and the number of points where the difference value was higher or lower than zero was used to show the relevant distribution. The authors’ image segmentation method is the only method used to calculate GVI in this comparative experiment. The authors extracted 390 random points at all sampling sites and used the five TSV configurations to obtain GVI results for those sampling sites. 

**Figure 12:**
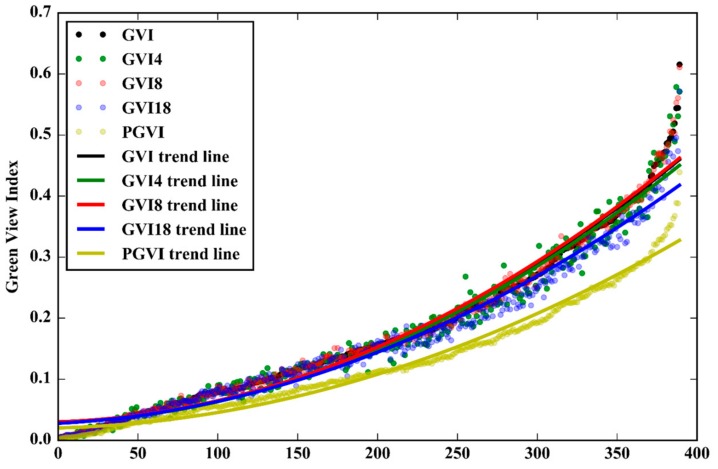
The green view index (GVI) values calculated by different methods.

The results showed that the GVI values obtained using the five TSV configurations have varying degrees of difference. Figure 12 shows that the GVI results of the five different TSV configurations have the same increasing trend. Table 3 shows the average difference value and RMSE of GVI for the four comparative groups, and the number of GVI difference values greater or less than zero. The table shows that the GVI–PGVI group has the largest average difference value and RMSE among the groups, followed by the GVI–GVI18 group, the GVI–GVI4 group and the GVI–GVI8 group. The difference between GVI and GVI8 is the smallest among the groups, with 216 sampling sites where the difference is greater than zero and 171 sampling sites where it is less than zero. Therefore, under image resolution conditions equal to that of the TSV pictures, the difference between the GVI value calculated using six TSV pictures and that calculated using eight TSV pictures in the horizontal is the smallest. Conversely, the GVI value calculated using four TSV pictures exhibits the largest difference with that calculated using six TSV pictures, which could be caused by the limited coverage of the TSV pictures (see the first and second rows in Figure 11). The GVI values calculated from TSV panoramas differ considerably from those calculated via all other methods because the stitching procedure merges the same area marked by registration points between two adjacent TSV pictures. Therefore, the authors suggest that in the future, researchers use six horizontal TSV pictures with inter-picture angles of 60° to capture the street-level visual landscape. Moreover, using this method decreases data consumption cost by limiting the acquisition of TSV pictures, thereby saving the API quota, which is now limited by Tencent. 

To summarize, the PGVI values have the largest RMSE among the four methods, and the panoramic stitching procedure can reduce the values. This section compared the GVI values calculated by five different methods to allow researchers in the future to determine a suitable method for GVI calculation to fulfil their research objectives.

### 5.4. Limitations and Future Research

Considerable room for improvement remains in the design of this study. The accuracy of the image segmentation could be further improved, for example, in some instances the façade of green signs, buildings and green vehicles were misrecognized using the current algorithm. The authors plan to combine the current method with SegNet to get a more robust segmentation framework to further optimize the segmentation method in the future. Moreover, in addition to road length and width, other road and geometric parameters, such as elevation, gradient, slope, illumination, crown size and tree size on both sides of the roads, should be considered in future research. Limited by the data acquisition process, the authors did not take those variables into account in this study. Many studies have explored the relationship between urban street greenery and human social psychology. The study’s extensive TSV picture dataset and GVI quantification method could provide technical support to land-senses ecology [37,38]. The application of the GVI indicator and TSV pictures could be used to further this research and is worthy of future study.

## 6. Conclusions

To conclude, SVPs are available for evaluating the degree of street greenery in a megacity. The horizontal GVI value can be precisely calculated using the current technique. Some road segments with low GVI values can be identified on a map. In our study area, the overall street greenery conditions have room for improvement, especially in the area within the third ring road. Among the different road types, T1, T4 and T5 represent key targets for road greening. The street-level greenery design of these road types should be improved in Beijing. According to the study data, road length has a positive correlation with the GVI values of most road types. However, road width has positive and negative influences on the GVI value depending on the road type. These widespread patterns could be useful for urban street management and planning. While ensuring the accuracy of the GVI values, the authors tried to use the least number of TSV pictures in the calculation process to save web resources and avoid redundant calculation. Literature on the GVI remains scarce at present, especially street-level greenery studies in a metropolis, which are usually very complex. Therefore, the current study contributes to the literature on the assessment of street-level visual greenery in a highly urbanized metropolis with multiple road types.

## Figures and Tables

**Table 1:** Rules for vegetation segmentation based on the HSI (Hue, Saturation and Intensity) colour model and Tencent Street View (TSV) pictures.

Rules for vegetation segmentation based on the HSI colour model and TSV pictures
Comment: *hue*, *saturation*, and *intensity* are the three elements in the TSV picture
Comment: *vegetation* is the vegetation segmentation results
Step 1: Transform the colour space of the TSV images from RGB to HSI
Step 2: Separate *hue*, *saturation* and *intensity* from the transformed image
Step 3: Extract vegetation pixels from the image
for each *pixel* [*i*, *j*] in *hue*:
if *hue* [*i*, *j*] > 75 *AND hue* [*i*, *j*] < 170:
Mark *hue* [*i*, *j*] as a green vegetation pixel *hue_mark_* [*i*, *j*]
Step 4: Reconstruct *hue_mark_*, *saturation* and *intensity* as a mask
Step 5: Mask out *vegetation* from the original image

**Table 2:** The correlation between road parameters and green view index (GVI) values of different road types.

	Road Type	Road Parameters			
		Width		Length	
Green View Index	T1	−0.332 **	(0.000)	0.263 **	(0.000)
	T2	−0.072	(0.250)	0.237 **	(0.000)
	T3	0.009	(0.845)	0.071	(0.110)
	T4	−0.207 **	(0.000)	0.206 **	(0.000)
	T5	0.121 **	(0.000)	0.241 **	(0.000)
	T6	−0.218 **	(0.000)	0.342 **	(0.000)
	T7	−0.089	(0.161)	0.296 **	(0.000)

** Pearson’s correlation coefficient is significant at the 0.000 level. Note: *p*-values (two-tailed) in parentheses.

**Table 3:** The difference values of four green view index (GVI) comparative groups.

	GVI and GVI4	GVI and GVI8	GVI and GVI18	GVI and PGVI
Average difference	0.0038	−0.0008	0.0122	0.0518
RMSE	0.0150	0.0058	0.0198	0.0636
Number of difference > 0	239	171	304	390
Number of difference < 0	151	216	86	0

**Figure A1:**
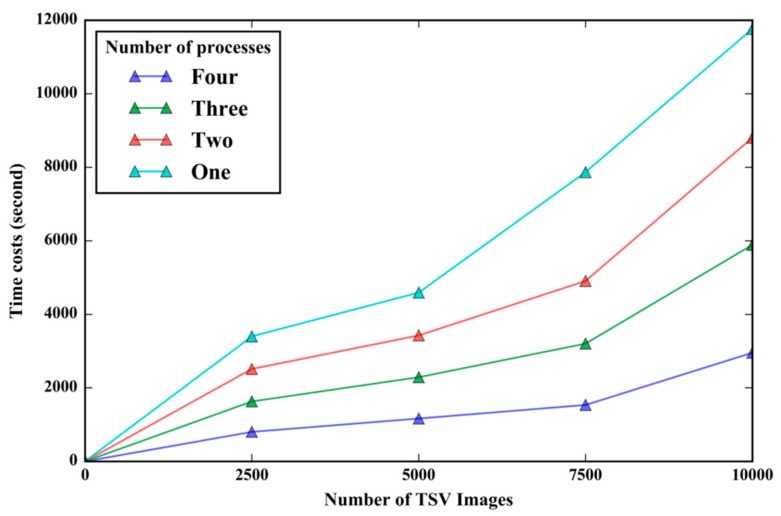
Comparison of the time costs of crawlers with different processes.

## Appendix A

### Appendix A.1. Parameters of Tencent Webservice API and Static Image API

In this study, we used two Tencent location services, Tencent WebService API and Tencent Static Image API. These APIs request services by sending server URL character strings. Table A1 shows the detailed URL parameters, their interpretations and examples of the Tencent WebService API. The WebService API was mainly used for searching nearest TSV pictures and metadata to connect road sample sites to TSVs’ ID in metadata. The “location” parameter was given the coordinates of road sample sites. The “radius” parameter was given the value of 50 m. The format of the metadata was JSON (JavaScript Object Notation). Table A2 shows the detailed URL parameters, their interpretations and examples of the Tencent Static Image API.

**Table A1:** Parameters of Tencent WebService API.

Parameters	Mandatory Field	Illustration	Example
id	Alternative	The unique identifier of one street scene.	id = 10011504120403141232200
location		Geographical coordinates; Location: latitude, longitude.	location = 41.057,116.636
poi		Information of street view images can be obtained by the corresponding ID of POI.	poi = 16459339205568630404
radius	Negative	The ids of street view image in specified radius; range: [0, 200], unit: meter.	radius = 50
output	Negative	The format of responding metadata. Default: JSON.	output = jsonp or json
callback	Negative	Callback function.	callback = function1
key	Affirmative	Developers’ key. (Application is free on Tencent location based service website)	key = DQTBZ-5VPHW-HDRRH-OJBIS-RSYQQ-NDF66

**Table A2:** Parameters of Tencent Static Image API.

Parameters	Mandatory Field	Illustration	Example
size	Affirmative	The pixel size of street view images. Width × length; Max value: 960 px, 640 px.	size = 600 × 600
location	Alternative	Geographical coordinates or address names.	location = Holiday Inn or location = 41.057,116.636.
pano		The unique identifier for each street scene.	pano = 10011010151015144953700
pitch	Negative	The angle of camera up or down; range: [−20, 90], default: 0.	pitch = 0
heading	Negative	The angle between camera and north direction; range: [0, 360], default: 0.	heading = 0
key	Affirmative	Developers’ key. (Application is free on Tencent location based service website)	DQTBZ-5VPHW-HDRRH-OJBIS-RSYQQ-NDF66

The second API was mainly used for acquiring TSV pictures from Tencent servers based on the mapping between road sample sites and their metadata. Tencent servers parsed URL character strings and extracted “size”, “pano/location”, “pitch”, “heading” and “key” parameters to locate the TSV picture, which was then sent to the user. In this study, the “size” parameter was set to 600 × 600, and the format of TSV pictures was set to PNG. Figure 4 in the article body shows URL examples and requesting results for these APIs.

These Tencent APIs have use limits. Every URL request sent to a server costs the quota of the Tencent developer key, which can be applied on the portal of the Tencent location service. To reduce pressure on the servers, Tencent has lowered the developer key quota from 100,000 times/IP/day to 10,000 times/IP/day, and the concurrent access times were limited to 5 times/IP/second. Therefore, it is very important to limit the number of requests as much as possible. Moreover, these two APIs’ quotas are independent and can be used at the same time.

### Appendix A.2. TSV Images Crawler

We encapsulated the two Tencent APIs into Python scripts to automatically search and download the TSV pictures at all sample sites. The working principle of crawling scripts are as follows: first, URL character strings are stitched based on the format of parameters in Table A1 and aggregated into a list; second, the “urlopen” module in the built-in package “urllib” is used to parse every URL character string and obtain the metadata of the nearest TSV picture; third, the TSV metadata is mapped to geometric coordinates; fourth, the ID field in the metadata is extracted, and the ID and other parameters in Table A2 are stitched into URL strings; fifth, the URL character strings are aggregated into a URL list; finally, the URL list is parsed to download TSV pictures. In addition, our Python version is 3.5.2.

In the process of obtaining the final GVI values, two types of point loss occurred. (1) Some sample sites did not have corresponding TSV pictures within 50 m. (2) TSV pictures taken in the fall or winter were excluded because green vegetation in these images was rare. Therefore, we obtained TSV pictures from April to October 2014 in the area surrounded by the sixth ring road (including the sixth ring road), resulting in a total number of TSV pictures of 55,539 × 6 = 333,234. Then, we used our segmentation program to extract green vegetation and to calculate the GVI value at all remaining sample sites.

### Appendix A.3. Operational Efficiency of the Parallel Crawler

To improve the operational efficiency of the crawling script, we implemented a parallel crawler using the “Multiprocessing” module in Python. In this comparative experiment, we independently executed four crawler scripts, and let them acquire 10,000 TSV pictures randomly picked from the TSV dataset. Every time 2500 pictures were successfully downloaded, the program recorded the time. The final time costs are shown in Figure A1. The legend in Figure A1 indicates the physical cores used by the scripts; for example, the label four under “numbers of processes” means that the script used four CPU cores for crawling pictures. The CPU used in this study is an Inter i7-6700 with four physical cores. We found that the time costs ratio of those four scripts was close to 4:3:2:1, corresponding to one, two, three, and four processes. The average network speed was approximately 10 Mbps. Speed fluctuations occurred during the experiment, so the results provide a general estimate of the time costs but are not constant. Obviously, the four processes script can save approximately 75% of the time cost of the single process script. Therefore, the parallel crawling script can take full advantage of the computer hardware resources and save time. In our study, we used a four processes crawler to acquire the 333,234 TSV pictures, which took a total of approximately 27 h.
